# Common pathogenetic traits of atopic dermatitis and autism spectrum disorders, potential connections and treatments: trivial Th2 inflammation or much more?

**DOI:** 10.3389/fimmu.2023.1201989

**Published:** 2023-07-27

**Authors:** Lucia Peterle, Serena Sanfilippo, Alessandro Tonacci, Federica Li Pomi, Francesco Borgia, Sebastiano Gangemi

**Affiliations:** ^1^ School and Operative Unit of Dermatology, Department of Clinical and Experimental Medicine, University of Messina, Messina, Italy; ^2^ School and Operative Unit of Allergy and Clinical Immunology, Department of Clinical and Experimental Medicine, University of Messina, Messina, Italy; ^3^ Institute of Clinical Physiology, National Research Council of Italy (IFC-CNR), Pisa, Italy

**Keywords:** atopic dermatitis, autism spectrum disorders, vitamin D, Th17 cells, microbiota, inflammation, autism, behaviors disorders

## 1 Introduction

Data show that there is a connection between autism spectrum disorders (ASD) and atopic dermatitis (AD). Several correlation studies have supported this hypothesis, even if not all agree. It is already known that genetic and environmental factors collaborate with immune dysregulation and inflammation in the possible pathophysiological mechanisms shared by these two conditions (1). A new study on the prevalence rates of AD and ASD in countries around the world highlighted a cubic relationship between the two diseases, underlining that the correlation exists and probably there are multiple factors and pathways involved (2).

Schematically, the probable mechanisms implicated are: 1) genetic predisposition (ADRB2, GATA-3, MIF, BDNF, SHANK3…) and epigenetic regulation (miR-146a, miR-155…); 2) formation of autoantibodies against brain antigens (anti-MBP, anti-MAG); 3) brain damage caused by microglia local inflammation and activation of mast cells (with repercussions on Th1 and/or Th17); 4) maternal and neonatal vitamin D deficiency in AD and ASD patients (with repercussions on T reg); 5) defect of hematoencephalic membrane due to the proinflammatory cytokines rise (TNF-α e IL-6) caused by microbiota alterations or recurrent S. aureus infections (with repercussions on Th1 and/or Th17) (3). Since the first point has been more extensively indagated in literature, in this work, we will discuss the other points and delve deeper into the most relevant results presented in the literature.

## 2 Discussion

Shin et al., in 2021 performed a study by creating a standard mouse model of ASD (offspring of pregnant BALB/c hairless mice treated with valproic acid) and by measuring some cytokines in blood, skin and brain samples at several weeks after birth. They observed: mast cells proliferation and activation; an altered lipid composition similar to AD; skin inflammation and subsequent emergence of neuroinflammation; increase in TNF-α and IL-17A especially in skin samples and increase in IFN-γ both in skin and in brain samples (responsible for toxicity and chronic inflammation); high values of Th2 cytokines (IL-4, IL-5, IL-13) especially in the first days after birth with subsequent persistence of high values mainly in the skin.

From these data it could be deduced a damage linked to Th2 cytokines that overcome the weak blood brain barrier of newborns with consequent neuroinflammation or a direct damage of microglia and neurons mediated by the neurotoxin IL-17A. INF-γ would seem to mediate the alteration of the skin and brain lipid composition (4).

A recent meta-analysis identified some cytokine [IL-6, IL-1β, IL-12p70, macrophage migration inhibitory factor (MIF), eotaxin-1, monocyte chemotactic protein-1(MCP-1), IL-8, IL-7, IL-2, IL-12, tumor necrosis factor-α (TNF-α), IL-17, IL-4] alterations as the main biomarkers of ASD (5).

These cytokines are the same ones involved in AD, so it is possible to speculate that a variation of the levels of these cytokines in the first years of life may predispose to the subsequent manifestation of ASD. Until now, the role of Th2 cytokines as a possible link between the two pathologies has been investigated but it seems increasingly plausible that other types of inflammation are also involved such as Th1, Th17, Treg.

In both ASD and AD, alterations of microbiota are common. ASD children with gastrointestinal symptoms also have immune imbalances. In these children, an increase in the production of pro-inflammatory cytokines IL-6 and TNF-α and a decrease in regulatory cytokines such as IL-10 were found, which may reflect an abnormal response to bacteria or food antigens. Gut microbiota regulates the transformation of naïve T cells into different types of Th cells such as Th1, Th2, and Th17 or Forkhead box P3 (Foxp3)+ Treg cells (6), suggesting that a dysbiosis could lead to the imbalance of these cell and related cytokines found in AD and ASD (7), as confirmed by the fact that intestinal dysbiosis precedes the onset of AD.

A recent study focused on the alteration of Tregs and Th17 subpopulations in ASD children. According to this study, in ASD peripheral blood cells there is a significant decrease in Tregs cells and an increase in the Th17 cells. It is the author’s opinion that the decrease in Tregs and increase in Th17 found may be related to microbial dysbiosis.

As such, a specific alteration of T clones in PBMCs of autism patients versus typically developing children had already been observed in previous studies: a reduction in Foxp3 (T reg) and an increase in STAT3 and ROR γ t (Th17), T-bet (Th1) and GATA-3 (Th2) were noted. Foxp3+ induced self-tolerance, so they prevent autoimmunity; Th2 are involved in neurodevelopment and GATA-3 regulates the synthesis of neurotransmitters and cell differentiation; Th1 play a role in neurological disorders (multiple sclerosis and depression) and regulate neuroinflammation together with Th17 (8).

Some of these cytokines performed fundamental functions in CNS: IL-1β in learning and synapse formation, IL-4 in neurogenesis and spatial learning, IL-17A in synaptic plasticity and in the management of anxiety (9).

Vitamin D plays a fundamental role in reducing inflammation, upregulating Treg cells and IL-10 production, therefore its supplementation, especially in deficient states, has been postulated as a possible treatment with promising results for autistic children and children with atopic dermatitis (10).

In fact, Vitamin D supplementation increases the number of Tregs and decreases the release of IL-6 and TNF-α, through the rise of IL-10 levels, contributing to a tolerogenic state. It is also seen that vitamin D3 levels is negatively correlated with anti-myelin associated glycoprotein (anti-MAG) levels and to ASD severity (11).

Another study evaluates the hypothesis that IL-17A can interfere with neuronal and behavioral development. They studied the brain changes that occur based on the age of exposure to IL-17 in murine models (embryonic *vs* adult brain) and ASD manifestation.

It has been demonstrated that the IL-17A produced by Th17 cells of the maternal intestine and placenta crosses the blood-placental barrier and embryonic brain development. In addition, it has been demonstrated that direct injection of IL-17A into the primary somatosensory cortex dysgranular zone (S1DZ) restores the ASD phenotypes of repetitive behavior or sociability. IL-17A also controls the gut microbiota, and its disruption causes autoimmunity.

Therefore, they proposed to treat pregnant psoriasis patients with anti-IL-17 (secukinumab) and evaluate the incidence of ASD in the offspring, if the incidence rate is reduced this could prove a relationship between MIA (maternal immune activation) and ASD through IL-17 signaling (12).

The potential pathogenetic role of Th17 is also known in AD. In a meta-analysis, the authors observed that Th17 cells and serum IL-17 levels are higher in AD patients than in controls. IL-17 has pro-inflammatory activity and it participates in the autoimmune response (13).

**Figure 1:**
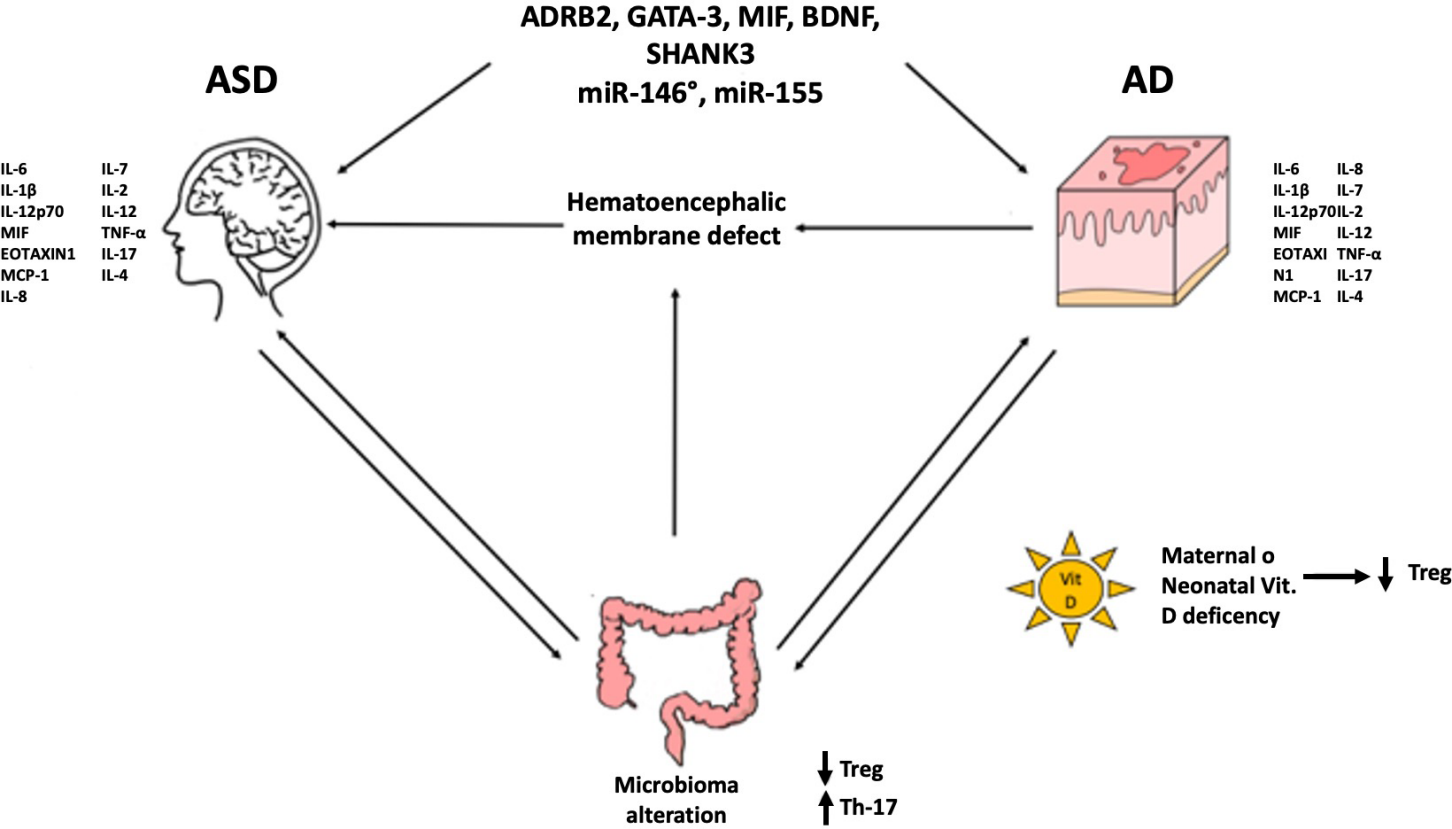
Possible common factors involved in the pathogenesis of ASD and AD.

An almost certain connection between these two disorders seems evident as shown in **Figure 1**.

Wan et al., in 2023, evaluated the correlation between AD and various neuropsychiatric disorders (autism, ADHD, anxiety, depression, obsessive-compulsive disorder,…), conducting a population-based cohort study on children under the age of 18. Although a strong association between AD and ASD has not been found in this population, an increased risk of developing autism was observed in patients with moderate AD or younger than 12 years of age, compared to controls. This suggests to the authors that the relationship between the two diseases may be due to several factors (including degree of disease and age of onset), in agreement with our hypothesis (14). Correlation between the two diseases seems to exist, but better targeting of the study population is needed so that interference can be reduced. Selecting a sample with specific characteristics (degree of disease severity, age of patients) could help to conduct more focused studies so that any correlation between the two diseases can be studied more appropriately.

We hypothesize the existence of a particular AD phenotype that predisposes to the development of ASD, so being able to identify the cytokine pathways involved between the two conditions could be useful to clarify this link. It could become the keystone to design a prompt treatment of this AD subgroup and prevent the onset of ASD.

We suggest that subsequent clinical studies should be designed for better defining the target population and dosing the main cytokines that seem relevant up to now (TNF-α, IL-1β, IL-6, INF-γ, IL-17, IL-12p70, MIF, IL-4 and IL-13). The results discussed, offering the basis for future research, should make clinicians, especially neuropsychiatrists and dermatologists, more aware of the possibility of an interconnection between these two pathologies resulting in a global assessment of the patient with autism.

### 2.1 Highlights

• AD specific phenotype could predispose to ASD.

• Identifying cytokine pathways clarifies the AD-ASD link, relevant cytokines are: TNF-α, IL-1β, IL-6, INF-γ, IL-17, IL-12p70, MIF, IL-4, IL-13.

• Further studies are needed to design a treatment of this AD subgroup and prevent the onset of ASD.

• Neuropsychiatrists and dermatologists should be aware of the possibility of an interconnection between these two conditions.

## Author contributions

Literature search: AT, LP, SS. Conceptualization: FB, FLP, SG. Study selection: LP, SS, AT. Manuscript drafting: LP, SS. Critical revision: SG, FB. Approval of the manuscript: all authors.

## References

[1] Billeci L Tonacci A Tartarisco G Ruta L Pioggia G Gangemi S. Association between atopic dermatitis and autism spectrum disorders: A systematic review. Am J Clin Dermatol (2015) 16(5):371–88. doi: 10.1007/s40257-015-0145-5 26254000

[2] Tonacci A Pioggia G Gangemi S. Autism spectrum disorders and atopic dermatitis: a new perspective from country-based prevalence data. Clin Mol Allergy (2021) 19:27. doi: 10.1186/s12948-021-00166-5 34930274 PMC8691085

[3] Tsai TY Chao YC Hsieh CY Huang YC. Association between atopic dermatitis and autism spectrum disorder: A systematic review and meta-analysis. Acta Derm Venereol (2020) 100:adv00146. doi: 10.2340/00015555-3501 32399577 PMC9137390

[4] Shin KO Crumrine DA Kim S Lee Y Kim B Abuabara K. Phenotypic overlap between atopic dermatitis and autism. BMC Neurosci (2021) 22(1):43. doi: 10.1186/s12868-021-00645-0 34157971 PMC8218496

[5] Zhao H Zhang H Liu S Luo W Jiang Y Gao J. Association of peripheral blood levels of cytokines with autism spectrum disorder: A meta-analysis. Front Psychiatry (2021) 12:670200. doi: 10.3389/fpsyt.2021.670200 34276441 PMC8283413

[6] Kim JE Kim HS. Microbiome of the skin and gut in atopic dermatitis (AD): understanding the pathophysiology and finding novel management strategies. J Clin Med (2019) 8(4):444. doi: 10.3390/jcm8040444 30987008 PMC6518061

[7] Saeed NK Al-Beltagi M Bediwy AS El-Sawaf Y Toema O. Gut microbiota in various childhood disorders: Implication and indications. World J Gastroenterol (2022) 28(18):1875–901. doi: 10.3748/wjg.v28.i18.1875 PMC9150060 35664966

[8] Ahmad SF Zoheir KMA Ansari MA Nadeem A Bakheet SA Al-Ayadhi LY. Dysregulation of Th1, Th2, Th17, and T regulatory cell-related transcription factor signaling in children with autism. Mol Neurobiol (2017) 54(6):4390–400. doi: 10.1007/s12035-016-9977-0 27344332

[9] Ellul P Rosenzwajg M Peyre H Fourcade G Mariotti-Ferrandiz E Trebossen V. Regulatory T lymphocytes/Th17 lymphocytes imbalance in autism spectrum disorders: evidence from a meta-analysis. Mol Autism (2021) 12(1):68. doi: 10.1186/s13229-021-00472-4 34641964 PMC8507168

[10] Infante M Sears B Rizzo AM Mariani Cerati D Caprio M Ricordi C. Omega-3 PUFAs and vitamin D co-supplementation as a safe-effective therapeutic approach for core symptoms of autism spectrum disorder: case report and literature review. Nutr Neurosci (2020) 23(10):779–90. doi: 10.1080/1028415X.2018.1557385 30545280

[11] Wang J Huang H Liu C Zhang Y Wang W Zou Z. Research progress on the role of vitamin D in autism spectrum disorder. Front Behav Neurosci (2022) 16:859151. doi: 10.3389/fnbeh.2022.859151 35619598 PMC9128593

[12] Fujitani M Miyajima H Otani Y Liu X. Maternal and adult interleukin-17A exposure and autism spectrum disorder. Front Psychiatry (2022) 13:836181. doi: 10.3389/fpsyt.2022.836181 35211045 PMC8861354

[13] Mao Y Yang C Tang L Liu G Cheng L Chen M. Increased expression of T helper 17 cells and interleukin-17 in atopic dermatitis: a systematic review and meta-analysis. Ann Palliat Med (2021) 10(12):12801–9. doi: 10.21037/apm-21-3590 35016438

[14] Wan J Shin DB Syed MN Abuabara K Lemeshow AR Gelfand JM. Atopic dermatitis and risk of major neuropsychiatric disorders in children: A population-based cohort study. J Eur Acad Dermatol Venereol (2023) 37(1):114–22. doi: 10.1111/jdv.18564 PMC9929490 36018560

